# Supplementary material for: Multiple mechanisms mediate the suppression of motion vision during escape maneuvers in flying Drosophila
Source: iScience. 2022 Sep 16;25(10):105143. doi: 10.1016/j.isci.2022.105143 (PMC9523382; doi:10.1016/j.isci.2022.105143)
Supplement: Document S1. Figures S1–S3 [file mmc1.pdf]

iScience, Volume *25*

Supplemental information

Multiple mechanisms mediate the suppression of motion vision during escape maneuvers in flying *Drosophila*

Philippe Jules Fischer and Bettina Schnell

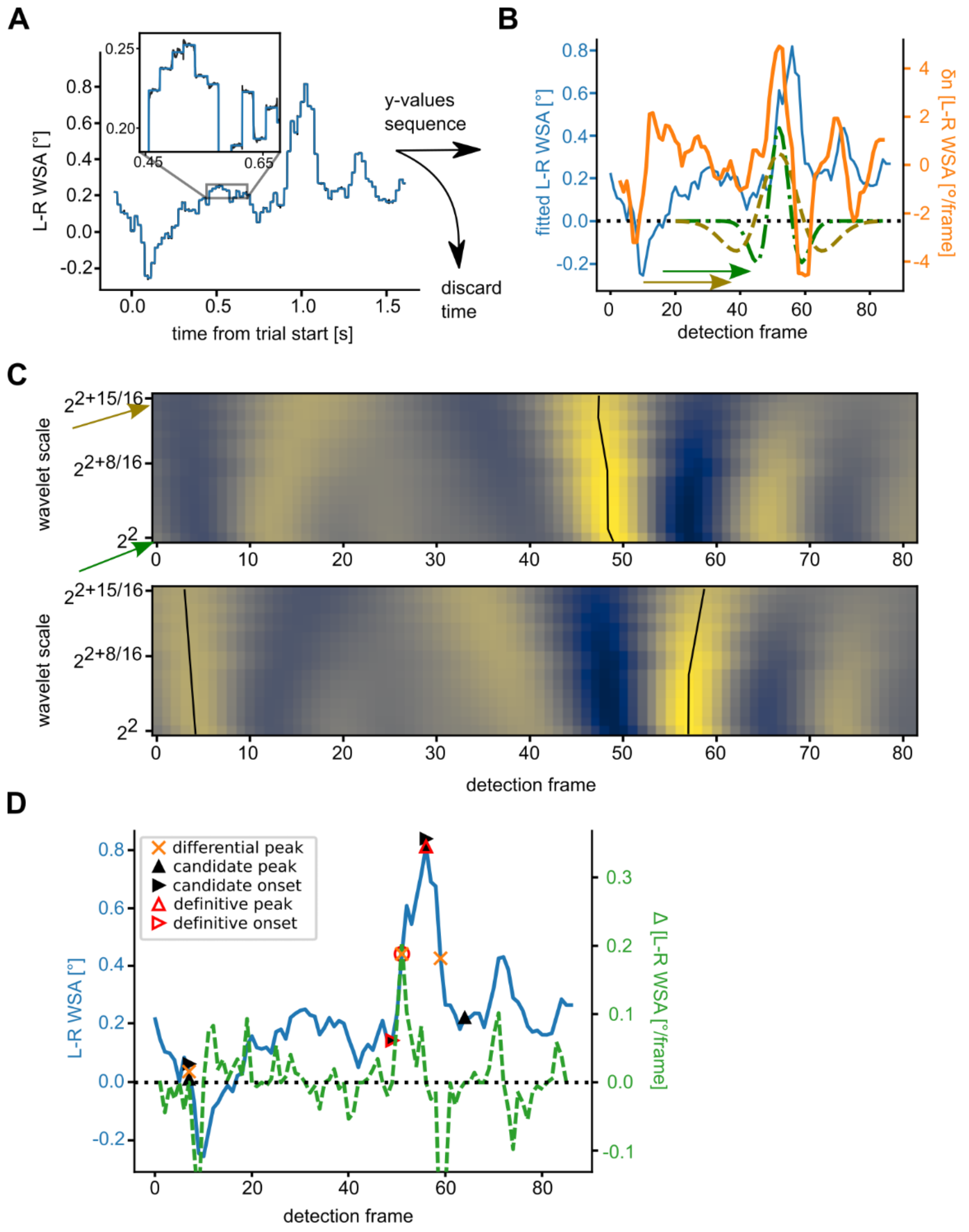

Figure S1

Illustration of the saccade detection algorithm, related to STAR Methods. (**A**) The wingbeat amplitude signal is corrupted by additive noise, which persists in the L-R WSA (black). Using the prior knowledge of approximate camera frame rate, a piece-wise-constant function is fitted to it (blue), to retrieve a distortion-free downsampled signal as a sequence of y-values. (**B**) Y-values form a continuous-value time-series (blue), which is differentiated using the central difference theorem (orange). Wavelet functions of the Ricker (derivative of a gaussian) family are applied as filters for peaks in the derived signal with scales between 4 (green) and 8 (brown) similar to a convolution. (**C**) Exemplary result of continuous wavelet transform (CWT) in the given range. Top shows CWT of positive signal, bottom is CWT of negative, arrows point at scales of wavelets in (B). Ridges across wavelet scales correspond to sharp transients, which are candidates for saccades. Rightward saccades correspond to peaks in the positive signal, and leftward saccades to peaks in the negative. (**D**) Ridge locations in CWT in positive and negative signals are treated as candidate peaks and additional post-selection criteria used to identify sufficiently prominent, stand-alone peaks in L-R WSA as saccades. Saccade onset and peak times are identified as time points closest to zero in the simple (backward difference) derivative.

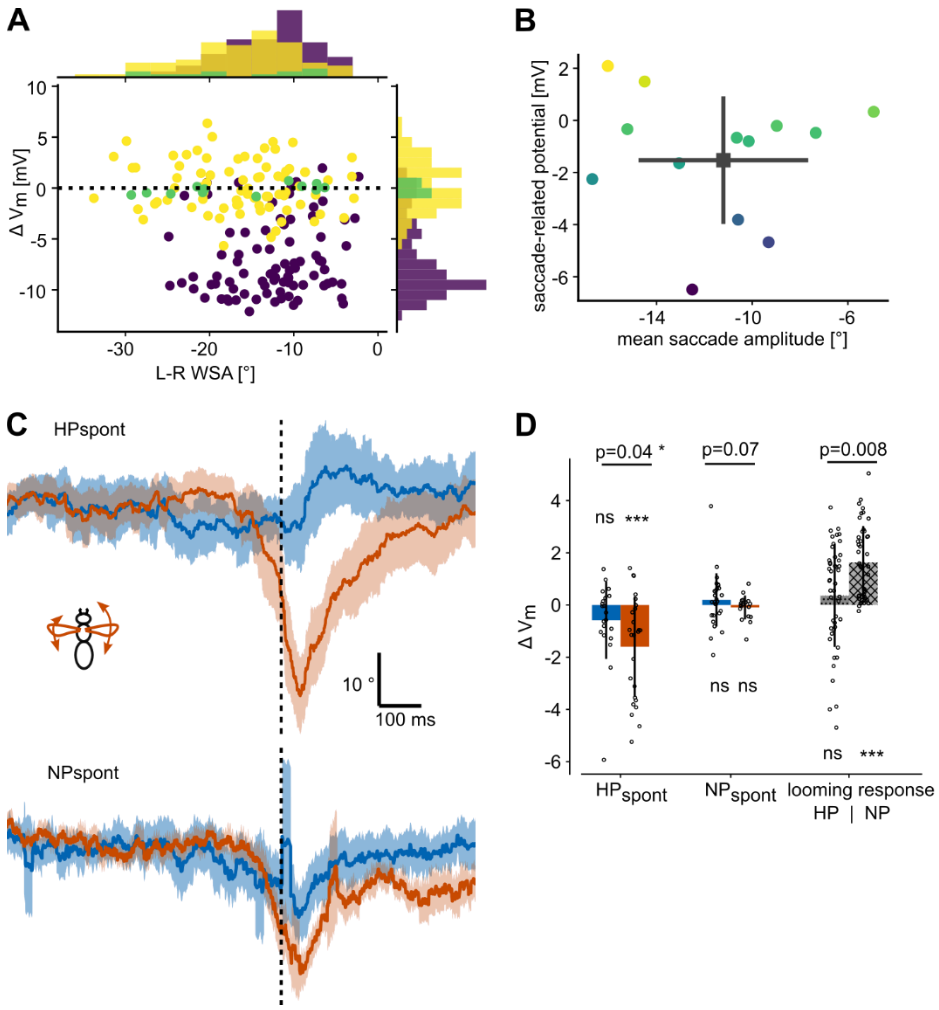

Figure S2

Quantitative analysis of HS cell responses during saccades to the left, related to Figure 2. (**A**) Voltage deviation from baseline (ΔV_m) against L-R WSA for all spontaneous saccades of three exemplary HS cells from Fig. 2D color-code accordingly (violet: hyperpolarizing cell, yellow: depolarizing cell, green: no change in polarization). (**B**) Mean ΔV_m plotted against mean L-R WSA for each recorded cell during spontaneous saccades to the left (color-coded as in Fig. 2C, D). (**C**) Mean ± s.e.m. of L-R WSA of the HP_{spont} subgroup (top, N = 6 flies) and the NP_{spont} subgroup (N = 5 flies) corresponding to Fig. 2E and F (saccade trials shown in blue, non-saccade trials in red). (**D**) Mean ΔV_m during trials with (red) or without (blue) looming-elicited saccades (individual trials are indicated by circles) for the HP_{spont} and NP_{spont} subgroups corresponding to Fig. 2E and F. A two-sided Wilcoxon test was performed to test whether the distributions are significantly different from zero, a one-sided Mann-Whitney-U to test for saccade-dependent differences. Responses to the ipsilateral looming stimulus itself (indicated by grey bars and circles) are significantly different between HP_{spont} (gray dotted) and NP_{spont} (gray hatched) groups ($p = 0.0081$) with the latter being significantly different from zero ($p = 1.0*10^{-8}$).

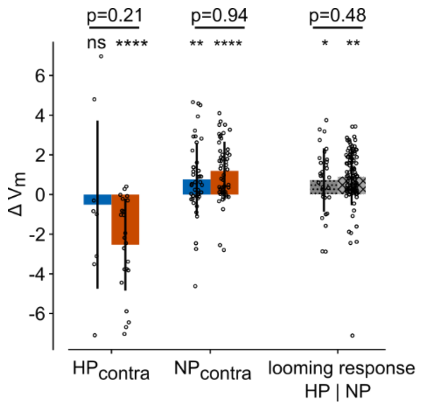

Figure S3

Quantitative analysis of response to contralateral looming, related to Figure 4. Mean ΔV_m during trials with (red) or without (blue) looming-elicited saccades (individual trials are indicated by circles) for the HP_{contra} and NP_{contra} subgroups corresponding to Fig. 4D and E. A two-sided Wilcoxon test was performed to test whether the distributions are significantly different from zero, a one-sided Mann-Whitney-U to test for saccade-dependent differences. The looming stimulus significantly depolarizes all cells without significant difference between HP_{contra} (gray dotted, $p = 0.015$) and NP_{contra} (gray hatched, $p = 1.6*10^{-10}$) subsets.
